# Surgical Principles and Diseases of the Face, Mouth and Jaws

**Published:** 1902-10

**Authors:** 


### Books and Magazines.

---

SURGICAL PRINCIPLES AND DISEASES OF THE FACE, MOUTH AND JAWS.—A text-book of the surgical principles and surgical diseases of the face, mouth and jaws. For dental students. By H. Horace Grant, A. M., M. D., Professor of Surgery and of Clinical Surgery, Hospital College of Medicine; Professor of Oral Surgery, Louisville College of Dentistry, Louisville. Octavo volume of 231 pages, with 68 illustrations. Philadelphia and London: W. B. Saunders & Co. 1902. Cloth, $2.50, net.

Dr. Grant has prepared in this a text-book that will be invaluable to dental students and perhaps to beginners in medicine. It will be found to be a safe guide in all operative procedures in which a dentist is likely to be called upon to take in hand. The volume is well illustrated and is written in clear, concise language, admitting of no doubt in any instance as to the author's meaning.

